# The influence of health policy on early diagnosis and surgical incidence of developmental dysplasia of the hip

**DOI:** 10.1371/journal.pone.0200995

**Published:** 2018-07-30

**Authors:** Chia H. Chang, Yi-Ting Chiang, Likwang Chen, Ken N. Kuo

**Affiliations:** 1 Department of Pediatric Orthopedics, Bone and Joint Research Center, Chang Gung Memorial Hospital, Chang Gung University, Taoyuan, Taiwan; 2 Cochrane Taiwan, Taipei Medical University, Taipei, Taiwan; 3 Institute of Population Health Sciences, National Health Research Institutes, Zhunan, Taiwan; 4 Orthopedic Department, National Taiwan University Hospital, Taipei, Taiwan; Public Library of Science, UNITED KINGDOM

## Abstract

**Background:**

Hip screening is the standard approach for the early detection of developmental dysplasia of the hip (DDH). However, there is a lack of evidence regarding the effects of national policy on early diagnosis and later surgical incidence. The purpose of this national study is to estimate DDH incidence in the Taiwanese population through a new diagnosis definition and to examine whether a health promotion policy could reduce surgeries for DDH.

**Methods and results:**

Six birth-year cohorts (2000–2005) were evaluated for DDH diagnosis and related surgeries using the database of the National Health Insurance Administration, which covers 99% of the population of Taiwan. Children with three or more sequential International Classification of Disease, Ninth Revision (ICD-9) diagnosis codes (754.3x) in the outpatient claim file or DDH-related surgeries were studied. The outcome of hip screening was evaluated with the ratio of early diagnosis (0–6 months) to late diagnosis (1–5 years) and the incidence of major surgeries for DDH. DDH incidence was 1.54 per thousand live births (2,255/1,462,539). After a hip screening promotion policy was implemented in 2002, ratios of early/late diagnosis increased from 1.06, 1.25, 1.38, and 1.5 to 1.75 for the years 2000 to 2005, respectively. Incidences of major surgery decreased from 0.41–0.47 per thousand before policy administration to 0.33–0.37 per thousand after policy administration.

**Discussion:**

The DDH incidence of 1.54 per 1,000 in a geographically well-defined area offered epidemiological data for further studies in Asian populations. The results suggest that the health promotion policy is associated with an increase in early diagnosis and subsequently a decrease in surgeries for DDH.

## Introduction

Developmental dysplasia of the hip (DDH) is a common musculoskeletal disorder in infants. The general standard of this disease is early detection and early hip reduction, which can restore the disorder to normal in 90% of cases [1,2]. Late DDH diagnosis requires operations for profound dysplasia and dislocation of the hip [3]. In fact, even after a successful operation for late-diagnosed DDH, 46% of patients in a 45-year follow-up still required total hip replacement for osteoarthritis [4]. Evidence suggests that treatment for late-diagnosed DDH is unlikely to result in a normal hip, with this disease still leading to early degeneration and a long-term burden to the health care system.

Hip screening aims to prevent late diagnosis and related surgeries [5,6]. Therefore, incidence of surgery for DDH has served as an indicator of hip screening effectiveness [7–9]. Godward et al. used an orthopedic surveillance scheme and inpatient data to estimate an incidence of surgeries for DDH of 0.78 per 1,000 live births in the United Kingdom [7]. In South Australia, Chan et al. used birth defect registration and an inpatient database to report an incidence of DDH of 7.74 per 1,000 live births and an incidence of surgery of 0.46 per 1,000 live births [8]. Ultrasound hip screening in Germany led to a lower surgical incidence of 0.26 per 1,000 live births [9].

Hip screening in Taiwan was performed by pediatricians or primary physicians in newborn nurseries, mainly using the Barlow and Ortolani tests. Pediatricians and primary family doctors also checked the limitation of hip abduction and Galeazzi sign during the three well-baby check-ups in the first month, 2–3 months, and 4–10 months. Suspected cases were referred to orthopedic doctors. All medical expenses related to outpatient and inpatient services were reimbursed by single government payer via the National Health Insurance (NHI) service. The NHI service started in 1995 and has gradually been extended to 99% of the 23 million residents of Taiwan [10,11]. It has provided an opportunity to survey disease incidence and the effects of health policy [12].

In efforts to improve child health, the Health Promotion Administration in Taiwan provides free well-baby and well-child check-ups, including vaccinations, seven times before school age. The first four check-ups occur in the first 1.5 years of life. When the policy commenced in 2002, it was implemented throughout Taiwan instantaneously. The agency launched a policy to enhance early detection of congenital disorders such as DDH, hearing impairment, and undescended testis. The policy has been implemented through periodic instructional lectures for primary medical professionals throughout Taiwan. Additionally, case referral is facilitated by quick access to designated medical centers with pediatric orthopedic surgeons. Once diagnosis is confirmed, there is a monetary reward from the Health Promotion Administration to the primary physician. Nevertheless, the health promotion policy needs to be verified by a subsequent decrease in surgeries for DDH. This study reviewed DDH-related data in the NHI databank to answer the following questions: (1) Can incidence of DDH be estimated from national medical insurance data? (2) Does health policy increase early detection rate of DDH? (3) Does early detection result in a decrease of surgeries for DDH, especially major surgeries?

## Materials and methods

### Study design and setting

The study had the approval of the IRB at the Chang Gung Medical Foundation (201600345B0C601). The study consisted of a retrospective population-based cohort using the Taiwan NHI research databank from January 2000 to December 2010. Disease incidence and related treatment for DDH were analyzed among the six birth-year cohorts from 2000 to 2005 in order to develop a 5-year survey covering every live birth.

### Study subjects

**Fig 1:**
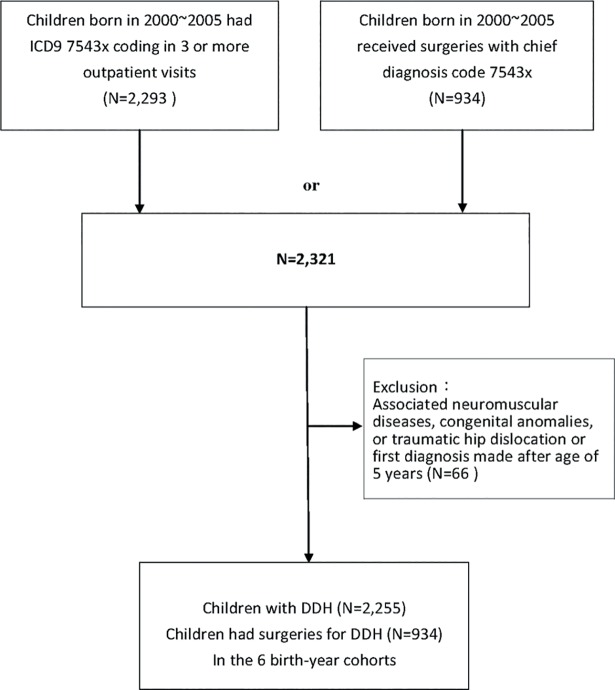
Flowchart of identification of study subjects.

Identity-scrambled data of 1,462,539 live births in the six birth-year cohorts (2000–2005) were reviewed according to the ICD-9 diagnosis code of 754.3x. Outpatient and inpatient cases were reconciled, duplications were removed, and the sums of cases were calculated. Cases with the 754.3x-associated diagnosis code of neuromuscular diseases, congenital anomaly, or acquired hip fracture/dislocation were excluded. For an even 5-year survey of the six birth-year cohorts, only cases with the first diagnosis during the first 5 years were included. In addition, only those cases with the 754.3x diagnosis code as the major diagnosis in three or more orthopedic outpatient visits were regarded as DDH and so included in the study (Fig 1).

### Incidence of DDH

The incidence of DDH was calculated by dividing the total cases in a given birth-year cohort by the total number of live births in that birth-year. The average incidence in the six birth-year cohorts was verified through comparison to a previously conducted newborn manual screening study in Taiwan [13].

### Early and late diagnosis

The diagnosis age was recorded during the first outpatient visit. Early diagnosis was defined as a diagnosis made in the first 6 months of life. Six months allowed a time lag between first detection/suspicion and diagnosis coding by orthopedic doctors, with treatment using a harness often being successful before 6 months of age. Late diagnosis was defined as a diagnosis made after 1 year of age, since these cases frequently require surgeries. Cases diagnosed between 6 months and 1 year served as a buffering gap between early and late diagnosis, thereby increasing the precision of the differentiation between these two groups. The ratio of early to late diagnosis was used to evaluate the quality of hip screening in each birth cohort year.

### Surgical incidence

Treatment using the following procedures was collected: closed reduction of the hip joint (79.75); open reduction of the hip joint (79.85); pelvic osteotomy (77.39 or 81.40); femoral osteotomy (77.25, 77.35, or 78.25); adductor tenotomy (83.12); or arthrogram examination (88.32) with the diagnosis code 754.3x. Cases with more than one surgery were identified by the first surgery. The number of surgical cases was grouped by infants’ birth-year. The incidence of surgery for DDH was calculated as the number of accumulated cases for one birth-year cohort divided by the total number of live births in that birth-year. A lower surgical incidence indicated a better outcome of national hip screening.

### Major surgery and minor surgery

Major surgeries for DDH included open reduction of the hip joint, pelvic osteotomy, and femoral osteotomy. Minor surgeries included closed reduction of the hip joint, adductor tenotomy, and arthrogram of the hip joint [7–9]. The decrease of surgical incidence, especially major surgeries, would indicate the success of early screening.

### Statistics

Since population sampling from the whole country was used, no adjustment or ascertainment procedure was required [8,9]. For comparison of the surgical rate in total DDH cases before and after policy administration, surgical DDH and non-surgical DDH in 2000–2002 and 2003–2005 were compared using a chi-square test. Data management and statistical analyses were performed using SAS version 9.2 and Microsoft Excel 2013 software.

## Results

Children with DDH in the six birth cohort years (2000–2005) were identified from the 2000–2010 database to ensure a minimum 5-year follow-up for each birth cohort year. Orthopedic doctors made coding for diagnoses in 97% of cases referred from newborn nurseries, pediatricians, obstetricians, and family doctors. In total, 2,255 DDH cases were diagnosed among the 1,462,539 live births in the six birth-year cohorts. DDH incidence averaged 1.54%, varying from 1.40 to 1.76 per 1,000 live births. Although total live births per year in Taiwan decreased from 305,000 to 205,000 over the 6 years, the incidence of DDH remained relatively stable for each year (Table 1). The resulting incidence of 1.54% was similar to that found in a newborn nursery screening study from 1988 by an orthopedic doctor in Taiwan [13].

**Table 1:** Number of DDH by year of birth and year of diagnosis. The accumulated cases in one birth-year cohort were divided by the total live births to calculate DDH incidence.

Birth year	Diagnosis year											Total DDH	Total live births*	Incidence^#^
	2000	2001	2002	2003	2004	2005	2006	2007	2008	2009	2010			
2000	184	149	97	21	8	5						464	305,312	1.52
2001		170	156	62	15	5	1					409	260,354	1.57
2002			211	121	72	17	10	5				436	247,530	1.76
2003				148	125	41	5	4	3			326	227,070	1.44
2004					164	96	55	8	7	1		331	216,419	1.53
2005						136	95	43	9	4	2	289	205,854	1.40

*Data from Department of Statistics, Ministry of the Interior, Taiwan

^#^ Incidence rate: 1/1,000 live births

Cases for each birth-year were stratified by age at the first diagnosis to calculate the incidence by diagnosis age. An increase of early diagnosis incidence (0.92%) in 2002 might be the direct result of health policy. The ratios of early diagnosis before 6 months of age to late diagnosis after 1 year of age were 1.06, 1.25, 1.38, 1.50, 1.50, and 1.75 for infants born in 2000, 2001, 2002, 2003, 2004, and 2005, respectively. The early/late diagnosis ratio increased across the study (Table 2).

**Table 2:** Incidence of DDH per thousand was stratified by the diagnosis age and the ratio of early diagnosis over late diagnosis from 2000 to 2005.

Birth year	Diagnosis age									Early / late ratio
	0–3m	4–6m	7–12m	1–2yr	2–3yr	3–4yr	4–5yr	< 6m (early)	> 1yr (late)	
2000	0.48	0.23	0.15	0.47	0.13	0.05	0.02	0.71	0.67	1.06
2001	0.53	0.23	0.20	0.46	0.12	0.02	0.02	0.76	0.61	1.25
2002	0.65	0.27	0.17	0.50	0.09	0.05	0.04	0.92	0.67	1.38
2003	0.45	0.28	0.23	0.40	0.06	0.01	0.02	0.73	0.48	1.50
2004	0.55	0.24	0.21	0.40	0.08	0.05	0.01	0.79	0.53	1.50
2005	0.61	0.19	0.14	0.33	0.08	0.02	0.02	0.81	0.46	1.75

A total of 934 DDH cases were surgically treated in the 1,462,539 live births of the six birth-year cohorts (0.64% in average). From these cases, 503 (54%) underwent surgeries at 1–2 years of age, the most common age requiring surgeries for late diagnosis. With an increasing ratio of early diagnosis, the surgical incidence decreased from 0.68%–0.71% in 2000–2002 to 0.49%–0.61% in 2003–2005. The rate of surgical DDH among total DDH was 570/1309 (44%) in 2000–2002 and 364/946 (38%) in 2003–2005 (*p* = 0.016, chi-square test). The significant decrease in the surgical rate after 2002 coincided with the increasing ratio of early diagnosis and the introduction of the policy (Table 3).

**Table 3:** Numbers of surgical cases stratified by operation age and the surgical incidence in the six cohorts.

Birth year	Operation age						Surgical DDH	Total live births	Surgical incidence
	0–6 m	7–12 m	1–2 yr	2–3 yr	3–4 yr	4–5 yr			
2000	28	33	110	25	8	5	209	305,312	0.68‰
2001	24	31	98	26	4	2	185	260,354	0.71‰
2002	22	22	97	20	9	6	176	247,530	0.71‰
2003	18	21	73	15	1	4	132	227,070	0.58‰
2004	22	19	72	12	4	3	132	216,419	0.61‰
2005	13	14	53	15	2	3	100	205,854	0.49‰
Total	127	140	503	113	28	23	934		

**Fig 2:**
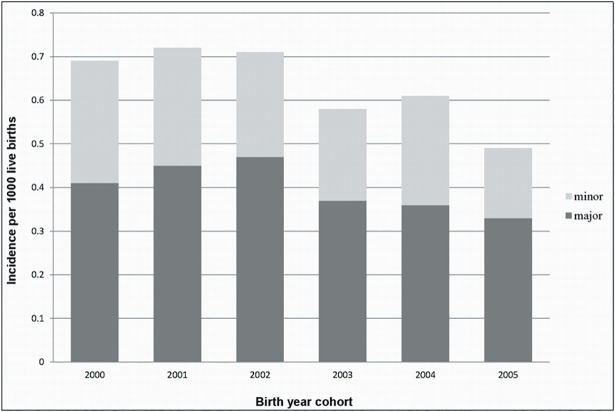
Decreasing incidence of surgery in the six birth-year cohorts.

From the total of 934 surgical cases, 587 children were treated by major surgeries, including open reduction, pelvic osteotomies, and femoral osteotomies. The distribution of major and minor surgeries was associated with operation age. The incidence of major surgeries decreased from 0.41%–0.47% in 2000–2002 to 0.33%–0.37% in 2003–2005 (Table 4) following the introduction of policy administration and an increase in early detection. The decreasing surgical incidences in the six birth-year cohorts mainly reflected the decrease in major surgeries (Fig 2).

**Table 4:** Distribution of major and minor surgeries by operation age revealed a strong association between surgical type and age. Incidence of major surgeries decreased gradually from 2002.

Birth year	Major surgeries / minor surgeries					Incidence of major surgeries in total live births
	0–6 m	7–12 m	1–2 yr	2–5 yr	Total	
2000	1/27	7/26	80/30	37/1	125/84	0.41‰
2001	2/22	4/27	78/20	32/0	116/69	0.45‰
2002	1/21	5/17	77/20	33/2	116/60	0.47‰
2003	1/17	6/15	58/15	20/0	85/47	0.37‰
2004	0/22	3/16	56/16	19/0	78/54	0.36‰
2005	2/11	3/11	43/10	19/1	67/33	0.33‰
Total	7/120	28/112	392/111	160/4	587/347	

## Discussion

As a country with a universal medical insurance system, Taiwan provides an excellent resource for characterizing disease incidence, rate of early diagnosis, and surgical incidence of DDH [12]. The National Health Insurance database included 99% of the 23 million population. The natural geographic border limited overseas treatment or additional cases from other countries. Despite an increase in 2002, the resulting incidence rates for the six birth-year cohorts were stable, possibly as a direct result of health policy. The DDH incidence of 1.54 per thousand live births provides an epidemiological starting point for further studies.

An unsolved problem in determining incidence of DDH in early infancy is that the diagnosis definition remains controversial [14]. Evidence from Barlow’s original paper in 1962 indicated that more than 80% of newborn hips with positive Barlow’s sign restored spontaneously within two weeks [15]. When neonatal instability of the hip was included in DDH, the incidence of DDH was magnified. Therefore, this study defined a case of DDH as a child who had three or more orthopedic clinic visits with a principal diagnosis code of 754.3, that is, a hip problem that drew more than three orthopedic evaluations or treatments. The resulting incidence of 1.54% was comparable to that found in the newborn nursery screening study referred to above [13]. Interestingly, Barlow reported a similar incidence of 1.55 per thousand in England after the first 2 weeks of life [15].

The screening method was the same before and after the policy. The policy enhanced medical professionals’ awareness of DDH and case referral. The increased case referral coincided with an abrupt increase in outpatient claims for DDH. The early diagnosis incidence of 0.92% in 2002 was higher than that of 0.76% in 2001 and 0.73% in 2003. The increasing trend in the early/late ratio may indicate medical care providers’ improved awareness of the need for hip screening. The effects of policy were maintained and subsequently (2003–2005) gradually strengthened (Table 2).

The overall goal of hip screening is to prevent surgery for late-diagnosed DDH and subsequent long-term comorbidity, such as osteonecrosis, residual dysplasia, and early hip degeneration [16,17]. This study revealed that the screening policy increased early diagnosis in the 2002 birth cohort and resulted in gradual decreases in surgical incidence in the birth cohorts from 2003–2005, especially of major surgeries. The data from a population of 1,462,539 live births in the 6-year period were statistically significant and sufficient to explain the dispute of a systematic literature review about hip screening [18].

This study has several limitations. First, the NHI database did not include information on case referrals. Therefore, the exact number of case referrals for DDH is unknown. Second, as the NHI databank contains no information on Pavlik harness prescriptions, the number of cases of a positive Ortolani test that required Pavlik harness was unknown.

As not all suspicious cases referred by primary physicians turned out to be positive upon consultation with pediatric orthopedic surgeons, there is a possibility that the policy resulted in over-diagnosis and over-treatment. However, in light of the long-term financial, medical, and social burdens of multiple surgeries and subsequent early degeneration in late-diagnosed DDH, we believe that promoting early diagnosis is an imperative standard despite the possible increased expenses, which should fall within a reasonable range.

## Conclusions

The incidence of DDH estimated from the population-wide databank was stationary over time in a geographically well-defined area and comparable to the results from a previous newborn screening study. Health promotion policy increased early diagnosis, subsequently resulting in a decrease of surgeries for DDH. These findings suggest a reliable measure of the incidence of DDH and of surgery for DDH from the data in a population-wide insurance databank and may further provide a definitive assessment of the quality of hip screening.

## Supporting information

S1 Text Tables and figures of DDH incidence by sex, age, area, and medical setting. (DOC)

S2 Text Tables and figures of surgeries for DDH by sex, age, operation type, and area. (DOC)
