# Supplementary material for: The influence of health policy on early diagnosis and surgical incidence of developmental dysplasia of the hip
Source: PLoS One. 2018 Jul 30;13(7):e0200995. doi: 10.1371/journal.pone.0200995 (PMC6066215; doi:10.1371/journal.pone.0200995)
Supplement: S1 Text — (DOC) [file pone.0200995.s001.doc]

Children had DDH diagnosis in outpatient visits between January 1, 1997 and December 31, 2010

(N=11,286)

Children received first DDH surgeries before 5 years old N=1,469

Inclusion criteria：

ICD 9 codes: 7543, 75430, 75431, 75432, 75433, 75434, 75435;

Exclusion criteria：

1. Patients only had <=2 DDH diagnosis, or
2. Patients never received DDH surgery

Children had DDH

N=3,990

Exclusion criteria:

1. Children born after 1 January 2006. (N=520)
2. Children had DDH after 5 years old (N=167)

Children had DDH before 5 years old and born before January 1, 2006

N=3,303

Figure 1 Selection of study subject for inclusion

Table 1、Case number of DDH patients by year of birth and year of diagnosis

| Birth year | Diagnosis year | | | | | | | | | | | |  |  | Total | Live births | incidence rate (per 1,000 live births) |
| --- | --- | --- | --- | --- | --- | --- | --- | --- | --- | --- | --- | --- | --- | --- | --- | --- | --- |
|  | 1997 | 1998 | 1999 | 2000 | 2001 | 2002 | 2003 | 2004 | 2005 | 2006 | 2007 | 2008 | 2009 | 2010 |  |  |  |
| 1997 | 33 | 109 | 99 | 31 | 13 | 11 | 0 | 0 | 0 | 0 | 0 | 0 | 0 | 0 | 296 | 326,002 | **0.91** |
| 1998 | 0 | 63 | 161 | 65 | 17 | 10 | 3 | 0 | 0 | 0 | 0 | 0 | 0 | 0 | 319 | 271,450 | **1.18** |
| 1999 | 0 | 0 | 180 | 139 | 79 | 14 | 14 | 7 | 0 | 0 | 0 | 0 | 0 | 0 | 433 | 283,661 | **1.53** |
| 2000 | 0 | 0 | 0 | 184 | 149 | 97 | 21 | 8 | 5 | 0 | 0 | 0 | 0 | 0 | 464 | 305,312 | **1.52** |
| 2001 | 0 | 0 | 0 | 0 | 170 | 156 | 62 | 15 | 5 | 1 | 0 | 0 | 0 | 0 | 409 | 260,354 | **1.57** |
| 2002 | 0 | 0 | 0 | 0 | 0 | 211 | 121 | 72 | 17 | 10 | 5 | 0 | 0 | 0 | 436 | 247,530 | **1.76** |
| 2003 | 0 | 0 | 0 | 0 | 0 | 0 | 148 | 125 | 41 | 5 | 4 | 3 | 0 | 0 | 326 | 227,070 | **1.44** |
| 2004 | 0 | 0 | 0 | 0 | 0 | 0 | 0 | 164 | 96 | 55 | 8 | 7 | 1 | 0 | 331 | 216,419 | **1.53** |
| 2005 | 0 | 0 | 0 | 0 | 0 | 0 | 0 | 0 | 136 | 95 | 43 | 9 | 4 | 2 | 289 | 205,854 | **1.40** |
| Total | 33 | 172 | 440 | 419 | 428 | 499 | 369 | 391 | 300 | 166 | 60 | 19 | 5 | 2 | 3303 |  |  |

**Table 1-1、Case number of DDH patients by year of birth and year of diagnosis (Girl)**

| Birth year | Diagnosis year | | | | | | | | | | | |  |  | Total | Live births | incidence rate (per 1,000 live births) |
| --- | --- | --- | --- | --- | --- | --- | --- | --- | --- | --- | --- | --- | --- | --- | --- | --- | --- |
|  | 1997 | 1998 | 1999 | 2000 | 2001 | 2002 | 2003 | 2004 | 2005 | 2006 | 2007 | 2008 | 2009 | 2010 |  |  |  |
| 1997 | 27 | 89 | 90 | 24 | 6 | 6 | 0 | 0 | 0 | 0 | 0 | 0 | 0 | 0 | 242 | 155,955 | **1.55** |
| 1998 | 0 | 48 | 133 | 55 | 9 | 6 | 2 | 0 | 0 | 0 | 0 | 0 | 0 | 0 | 253 | 129,988 | **1.95** |
| 1999 | 0 | 0 | 123 | 116 | 63 | 9 | 9 | 4 | 0 | 0 | 0 | 0 | 0 | 0 | 324 | 135,619 | **2.39** |
| 2000 | 0 | 0 | 0 | 137 | 119 | 75 | 16 | 3 | 4 | 0 | 0 | 0 | 0 | 0 | 354 | 145,586 | **2.43** |
| 2001 | 0 | 0 | 0 | 0 | 131 | 127 | 45 | 13 | 2 | 1 | 0 | 0 | 0 | 0 | 319 | 124,758 | **2.56** |
| 2002 | 0 | 0 | 0 | 0 | 0 | 156 | 105 | 57 | 12 | 6 | 4 | 0 | 0 | 0 | 340 | 117,993 | **2.88** |
| 2003 | 0 | 0 | 0 | 0 | 0 | 0 | 115 | 93 | 36 | 3 | 1 | 2 | 0 | 0 | 250 | 108,086 | **2.31** |
| 2004 | 0 | 0 | 0 | 0 | 0 | 0 | 0 | 124 | 79 | 49 | 6 | 7 | 0 | 0 | 265 | 102,780 | **2.58** |
| 2005 | 0 | 0 | 0 | 0 | 0 | 0 | 0 | 0 | 113 | 73 | 33 | 2 | 3 | 1 | 225 | 98,476 | **2.28** |
| Total | 27 | 137 | 346 | 332 | 328 | 379 | 292 | 294 | 246 | 132 | 44 | 11 | 3 | 1 | 2572 |  |  |

**Table 1-2、Case number of DDH patients by year of birth and year of diagnosis (boy**)

| Birth year | Diagnosis year | | | | | | | | | | | |  |  | Total | Live births | incidence rate (per 1,000 live births) |
| --- | --- | --- | --- | --- | --- | --- | --- | --- | --- | --- | --- | --- | --- | --- | --- | --- | --- |
|  | 1997 | 1998 | 1999 | 2000 | 2001 | 2002 | 2003 | 2004 | 2005 | 2006 | 2007 | 2008 | 2009 | 2010 |  |  |  |
| 1997 | 3 | 13 | 9 | 6 | 6 | 5 | 0 | 0 | 0 | 0 | 0 | 0 | 0 | 0 | 42 | 170,047 | **0.25** |
| 1998 | 0 | 10 | 27 | 9 | 6 | 4 | 1 | 0 | 0 | 0 | 0 | 0 | 0 | 0 | 57 | 141,462 | **0.40** |
| 1999 | 0 | 0 | 56 | 22 | 16 | 4 | 5 | 3 | 0 | 0 | 0 | 0 | 0 | 0 | 106 | 148,042 | **0.72** |
| 2000 | 0 | 0 | 0 | 46 | 26 | 21 | 4 | 5 | 1 | 0 | 0 | 0 | 0 | 0 | 103 | 159,726 | **0.64** |
| 2001 | 0 | 0 | 0 | 0 | 38 | 27 | 15 | 2 | 3 | 0 | 0 | 0 | 0 | 0 | 85 | 135,596 | **0.63** |
| 2002 | 0 | 0 | 0 | 0 | 0 | 54 | 16 | 13 | 5 | 4 | 1 | 0 | 0 | 0 | 93 | 129,537 | **0.72** |
| 2003 | 0 | 0 | 0 | 0 | 0 | 0 | 33 | 31 | 4 | 2 | 3 | 1 | 0 | 0 | 74 | 118,984 | **0.62** |
| 2004 | 0 | 0 | 0 | 0 | 0 | 0 | 0 | 36 | 17 | 5 | 2 | 0 | 1 | 0 | 61 | 113,639 | **0.54** |
| 2005 | 0 | 0 | 0 | 0 | 0 | 0 | 0 | 0 | 23 | 19 | 8 | 7 | 1 | 0 | 58 | 107,378 | **0.54** |
| Total | 3 | 23 | 92 | 83 | 92 | 115 | 74 | 90 | 53 | 30 | 14 | 8 | 2 | 0 | 679 |  |  |


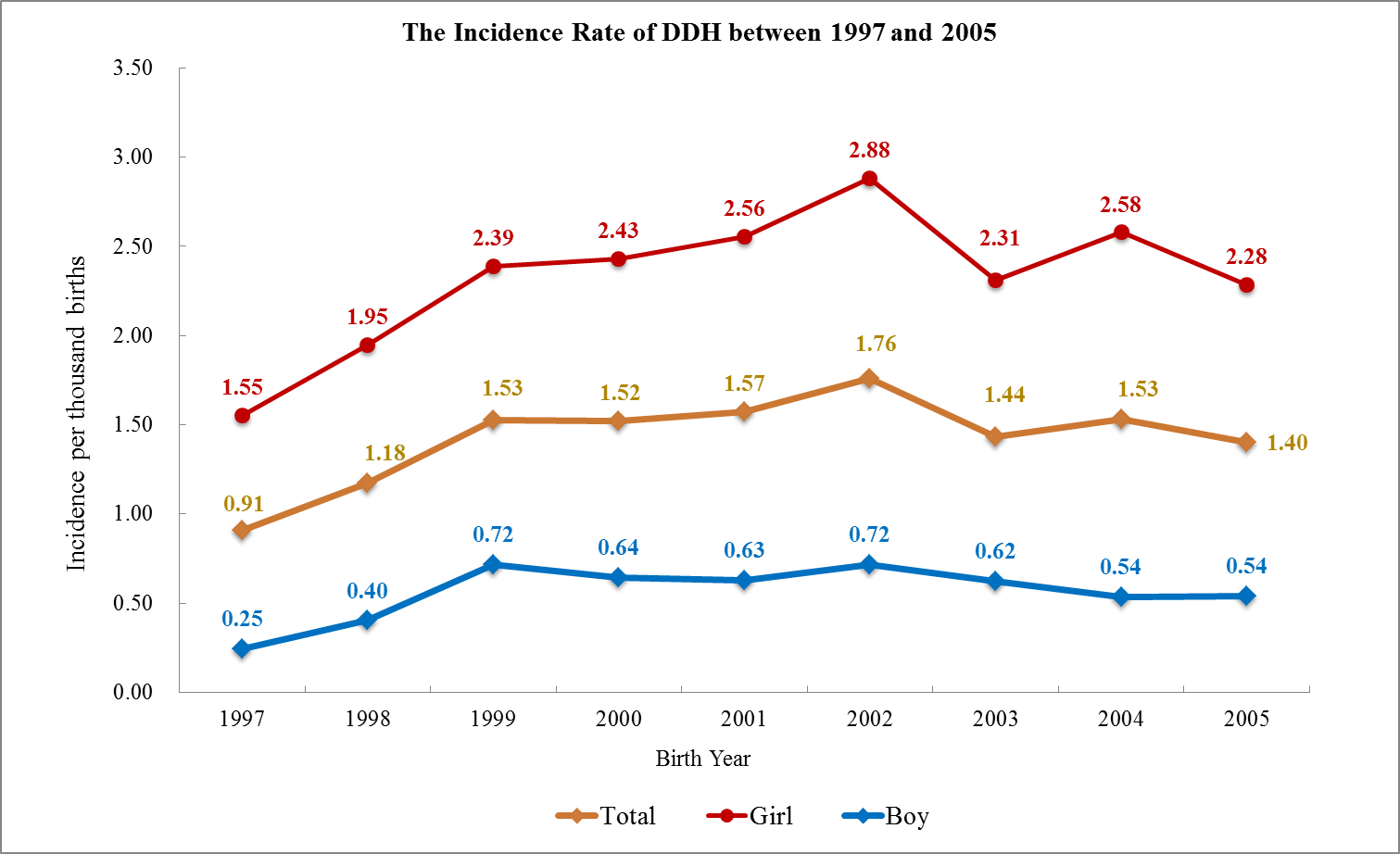


Table 2-1、Case number of DDH patients by year of birth and diagnosed age (Total)

| Birth year | Diagnosed age | | | | | | | | Total |
| --- | --- | --- | --- | --- | --- | --- | --- | --- | --- |
|  | 3 months | 4~6months | 7~9 months | 10~12months | 1~2 years | 2~3 years | 3~4 years | 4~5 years |  |
| 1997 | 17 | 13 | 13 | 13 | 157 | 42 | 24 | 17 | 296 |
| 1998 | 47 | 49 | 38 | 20 | 117 | 27 | 12 | 9 | 319 |
| 1999 | 167 | 52 | 27 | 16 | 124 | 19 | 13 | 15 | 433 |
| 2000 | 145 | 71 | 34 | 11 | 142 | 39 | 15 | 7 | 464 |
| 2001 | 138 | 60 | 38 | 14 | 119 | 30 | 5 | 5 | 409 |
| 2002 | 162 | 66 | 30 | 13 | 123 | 21 | 12 | 9 | 436 |
| 2003 | 101 | 64 | 36 | 15 | 90 | 13 | 2 | 5 | 326 |
| 2004 | 120 | 51 | 37 | 9 | 86 | 17 | 10 | 1 | 331 |
| 2005 | 126 | 40 | 18 | 10 | 68 | 17 | 5 | 5 | 289 |
| Total | 1023 | 466 | 271 | 121 | 1026 | 225 | 98 | 73 | 3303 |

Table 2-2、Incidence rate of DDH patients by birth year and diagnosed age (Total)

| Birth year | Diagnosed age | | | | | |  |  |
| --- | --- | --- | --- | --- | --- | --- | --- | --- |
|  | 3 months | 4~6months | 7~9 months | 10~12months | 1~2 years | 2~3 years | 3~4 years | 4~5 years |
| 1997 | 0.052 | 0.040 | 0.040 | 0.040 | 0.482 | 0.129 | 0.074 | 0.052 |
| 1998 | 0.173 | 0.181 | 0.140 | 0.074 | 0.431 | 0.099 | 0.044 | 0.033 |
| 1999 | 0.589 | 0.183 | 0.095 | 0.056 | 0.437 | 0.067 | 0.046 | 0.053 |
| 2000 | 0.475 | 0.233 | 0.111 | 0.036 | 0.465 | 0.128 | 0.049 | 0.023 |
| 2001 | 0.530 | 0.230 | 0.146 | 0.054 | 0.457 | 0.115 | 0.019 | 0.019 |
| 2002 | 0.654 | 0.267 | 0.121 | 0.053 | 0.497 | 0.085 | 0.048 | 0.036 |
| 2003 | 0.445 | 0.282 | 0.159 | 0.066 | 0.396 | 0.057 | 0.009 | 0.022 |
| 2004 | 0.554 | 0.236 | 0.171 | 0.042 | 0.397 | 0.079 | 0.046 | 0.005 |
| 2005 | 0.612 | 0.194 | 0.087 | 0.049 | 0.330 | 0.083 | 0.024 | 0.024 |


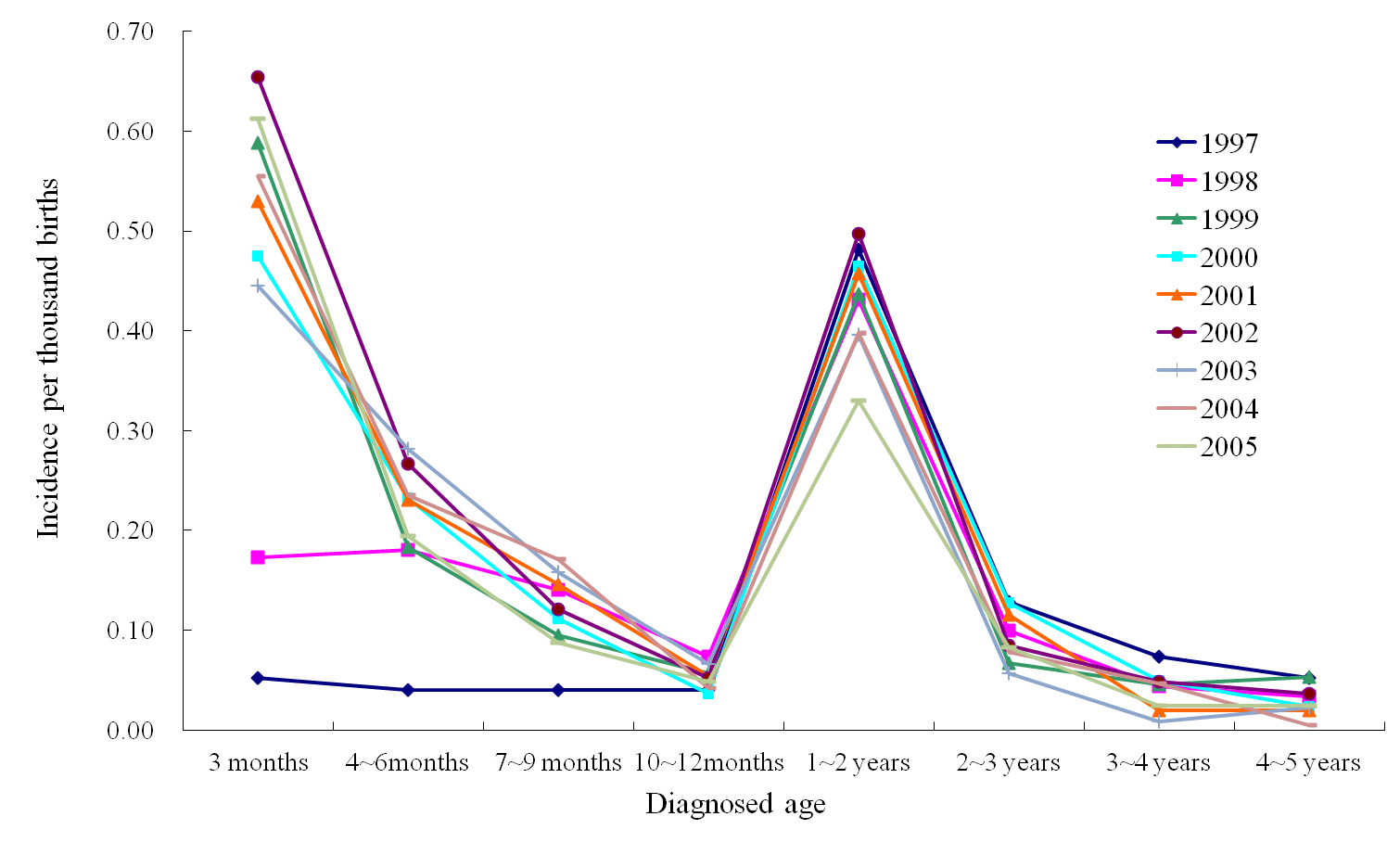


Table 2-1-1、Case number of DDH patients by year of birth and diagnosed age (Girl)

| Birth year | Diagnosed age | | | | | | | | Total |
| --- | --- | --- | --- | --- | --- | --- | --- | --- | --- |
|  | 3 months | 4~6months | 7~9 months | 10~12months | 1~2 years | 2~3 years | 3~4 years | 4~5 years |  |
| 1997 | 14 | 8 | 8 | 13 | 138 | 38 | 14 | 9 | 242 |
| 1998 | 35 | 39 | 33 | 17 | 99 | 18 | 6 | 6 | 253 |
| 1999 | 111 | 43 | 23 | 13 | 103 | 14 | 8 | 9 | 324 |
| 2000 | 109 | 50 | 31 | 6 | 117 | 27 | 10 | 4 | 354 |
| 2001 | 101 | 48 | 32 | 12 | 96 | 24 | 3 | 3 | 319 |
| 2002 | 124 | 49 | 23 | 11 | 102 | 18 | 6 | 7 | 340 |
| 2003 | 80 | 51 | 22 | 10 | 74 | 10 | 0 | 3 | 250 |
| 2004 | 94 | 39 | 25 | 7 | 75 | 16 | 9 | 0 | 265 |
| 2005 | 104 | 34 | 14 | 7 | 52 | 10 | 0 | 4 | 225 |
| Total | 772 | 361 | 211 | 96 | 856 | 175 | 56 | 45 | 2572 |

Table 2-2-1、Incidence rate of DDH patients by birth year and diagnosed age (Girl)

| Birth year | Diagnosed age | | | | | | | |
| --- | --- | --- | --- | --- | --- | --- | --- | --- |
|  | 3 months | 4~6months | 7~9 months | 10~12months | 1~2 years | 2~3 years | 3~4 years | 4~5 years |
| 1997 | 0.090 | 0.051 | 0.051 | 0.083 | 0.885 | 0.244 | 0.090 | 0.058 |
| 1998 | 0.269 | 0.300 | 0.254 | 0.131 | 0.762 | 0.138 | 0.046 | 0.046 |
| 1999 | 0.818 | 0.317 | 0.170 | 0.096 | 0.759 | 0.103 | 0.059 | 0.066 |
| 2000 | 0.749 | 0.343 | 0.213 | 0.041 | 0.804 | 0.185 | 0.069 | 0.027 |
| 2001 | 0.810 | 0.385 | 0.256 | 0.096 | 0.769 | 0.192 | 0.024 | 0.024 |
| 2002 | 1.051 | 0.415 | 0.195 | 0.093 | 0.864 | 0.153 | 0.051 | 0.059 |
| 2003 | 0.740 | 0.472 | 0.204 | 0.093 | 0.685 | 0.093 | 0.000 | 0.028 |
| 2004 | 0.915 | 0.379 | 0.243 | 0.068 | 0.730 | 0.156 | 0.088 | 0.000 |
| 2005 | 1.056 | 0.345 | 0.142 | 0.071 | 0.528 | 0.102 | 0.000 | 0.041 |


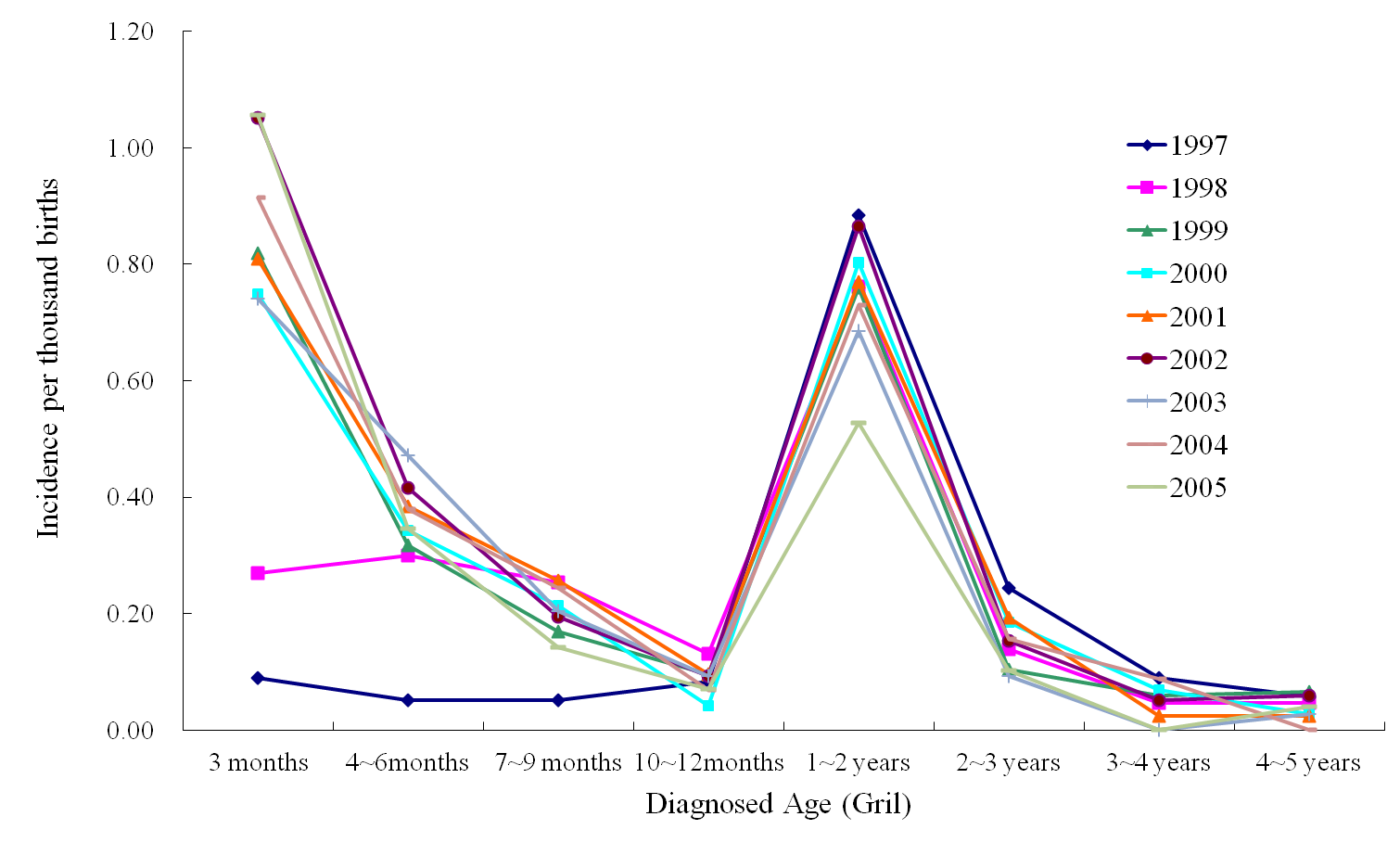


Table 2-1-2、Case number of DDH patients by year of birth and diagnosed age (Boy)

| Birth year | Diagnosed age | | | | | | | | Total |
| --- | --- | --- | --- | --- | --- | --- | --- | --- | --- |
|  | 3 months | 4~6months | 7~9 months | 10~12months | 1~2 years | 2~3 years | 3~4 years | 4~5 years |  |
| 1997 | 1 | 2 | 2 | 0 | 17 | 4 | 8 | 8 | 42 |
| 1998 | 8 | 10 | 5 | 2 | 16 | 8 | 5 | 3 | 57 |
| 1999 | 55 | 9 | 4 | 3 | 20 | 5 | 4 | 6 | 106 |
| 2000 | 36 | 19 | 3 | 5 | 21 | 12 | 4 | 3 | 103 |
| 2001 | 37 | 12 | 5 | 2 | 20 | 5 | 2 | 2 | 85 |
| 2002 | 37 | 17 | 7 | 2 | 19 | 3 | 6 | 2 | 93 |
| 2003 | 21 | 13 | 14 | 4 | 15 | 3 | 2 | 2 | 74 |
| 2004 | 26 | 10 | 10 | 2 | 10 | 1 | 1 | 1 | 61 |
| 2005 | 22 | 6 | 4 | 2 | 12 | 7 | 5 | 0 | 58 |
|  | 243 | 98 | 54 | 22 | 150 | 48 | 37 | 27 | 679 |

Table 2-2-2、Incidence rate of DDH patients by birth year and diagnosed age (Boy)

| Birth year | Diagnosed age | | | | | | | |
| --- | --- | --- | --- | --- | --- | --- | --- | --- |
|  | 3 months | 4~6months | 7~9 months | 10~12months | 1~2 years | 2~3 years | 3~4 years | 4~5 years |
| 1997 | 0.006 | 0.012 | 0.012 | 0.000 | 0.100 | 0.024 | 0.047 | 0.047 |
| 1998 | 0.057 | 0.071 | 0.035 | 0.014 | 0.113 | 0.057 | 0.035 | 0.021 |
| 1999 | 0.372 | 0.061 | 0.027 | 0.020 | 0.135 | 0.034 | 0.027 | 0.041 |
| 2000 | 0.225 | 0.119 | 0.019 | 0.031 | 0.131 | 0.075 | 0.025 | 0.019 |
| 2001 | 0.273 | 0.088 | 0.037 | 0.015 | 0.147 | 0.037 | 0.015 | 0.015 |
| 2002 | 0.286 | 0.131 | 0.054 | 0.015 | 0.147 | 0.023 | 0.046 | 0.015 |
| 2003 | 0.176 | 0.109 | 0.118 | 0.034 | 0.126 | 0.025 | 0.017 | 0.017 |
| 2004 | 0.229 | 0.088 | 0.088 | 0.018 | 0.088 | 0.009 | 0.009 | 0.009 |
| 2005 | 0.205 | 0.056 | 0.037 | 0.019 | 0.112 | 0.065 | 0.047 | 0.000 |


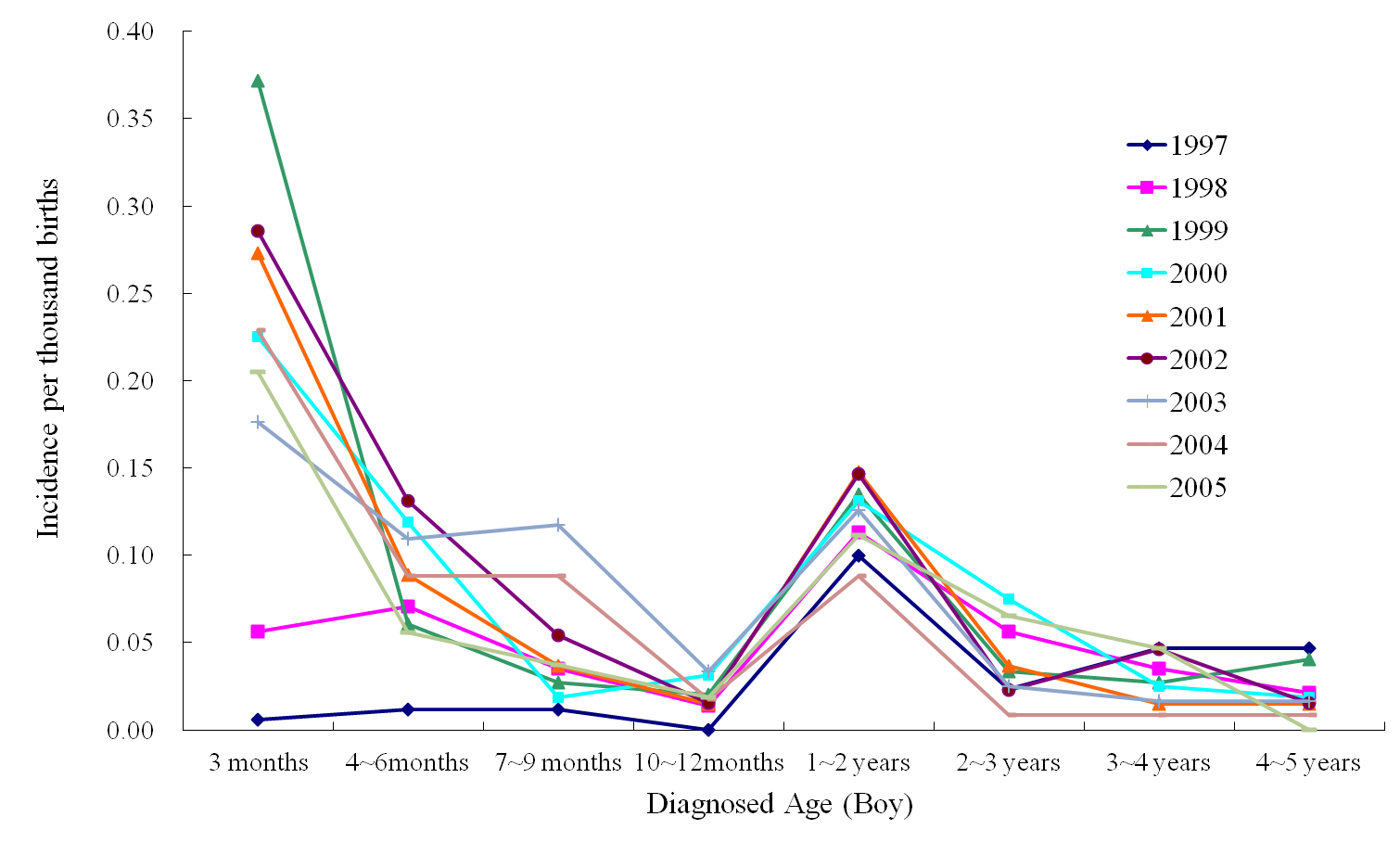


Table 3. Case number of DDH patients by year of birth and geographic area (Total)

|  | Northern | Central | Southern | Others | Total |
| --- | --- | --- | --- | --- | --- |
| 1997 | 153 | 72 | 68 | 3 | 296 |
| 1998 | 161 | 63 | 95 | 0 | 319 |
| 1999 | 147 | 79 | 204 | 3 | 433 |
| 2000 | 178 | 85 | 196 | 5 | 464 |
| 2001 | 159 | 71 | 174 | 5 | 409 |
| 2002 | 153 | 69 | 206 | 8 | 436 |
| 2003 | 121 | 45 | 158 | 2 | 326 |
| 2004 | 116 | 75 | 135 | 5 | 331 |
| 2005 | 98 | 52 | 138 | 1 | 289 |
| Total | 1286 | 611 | 1374 | 32 | 3303 |

Table 4. Case number of DDH patients by year of birth and medical setting (Total)

|  | Medical center | Regional hospital | District hospital | Clinics and others | Total |
| --- | --- | --- | --- | --- | --- |
| 1997 | 189 | 93 | 12 | 2 | 296 |
| 1998 | 205 | 101 | 9 | 4 | 319 |
| 1999 | 310 | 108 | 14 | 1 | 433 |
| 2000 | 348 | 86 | 28 | 2 | 464 |
| 2001 | 311 | 71 | 25 | 2 | 409 |
| 2002 | 345 | 74 | 16 | 1 | 436 |
| 2003 | 262 | 49 | 11 | 4 | 326 |
| 2004 | 254 | 56 | 18 | 3 | 331 |
| 2005 | 221 | 53 | 12 | 3 | 289 |
| Total | 2445 | 691 | 145 | 22 | 3303 |
