# Supplementary material for: The influence of health policy on early diagnosis and surgical incidence of developmental dysplasia of the hip
Source: PLoS One. 2018 Jul 30;13(7):e0200995. doi: 10.1371/journal.pone.0200995 (PMC6066215; doi:10.1371/journal.pone.0200995)
Supplement: S2 Text — (DOC) [file pone.0200995.s002.doc]

Inpatient visits which received surgery between January 1, 1997 and December 31, 2010

1997-2010 inpatient surgery

N=2,189

Outpatient visits which received surgery between January 1, 1997 and December 31, 2010

Inclusion criteria：(1) and (2)both match

(1)ICD 9 codes: 7543, 75430, 75431, 75432, 75433,75434, 75435;

(2)Order code: Major (64006B, 64064B, 64064C, 64171B, 64236B, 64240B, 64260B, 88038B, 88039B)

Minor(64073B, 64089B, 64193B, 64197B, 64197C, 88040B, 33063B, 64073C)

1997-2010 outpatient surgery

N=446

1997-2010 DDH surgeries

N=1,950

N=2,189

1997-2010 DDH surgeries

N=442

N=2,189

Exclusion criteria：

ICD 9 code: 270-279,330-337,341-343, 348,349,350-359,710,711,713,718, 730-732, 741, 742, 7553, 7556,756,758 Excluded 243 non-DDH surgeries

Children first received DDH surgeries

N=1,665

Children received first DDH surgeries before 5 years old and born before January 1, 2006

N=1,469

N=1,665

Exclusion criteria：

Children born after 1 January 2006. (N=136)

Children received surgeries after 5 years old (N=60)

7556,756,758 Excluded 243 non-DDH surgeries

Figure 1 Selection of study subject for inclusion

Table 1、Case number of first surgery by year of birth and year of operation

| Birth year | Operation year | | | | | | | | | | | |  |  | Total | Live births | incidence rate (per 1,000 live births) |
| --- | --- | --- | --- | --- | --- | --- | --- | --- | --- | --- | --- | --- | --- | --- | --- | --- | --- |
|  | 1997 | 1998 | 1999 | 2000 | 2001 | 2002 | 2003 | 2004 | 2005 | 2006 | 2007 | 2008 | 2009 | 2010 |  |  |  |
| 1997 | 21 | 85 | 64 | 17 | 7 | 1 | 0 | 0 | 0 | 0 | 0 | 0 | 0 | 0 | 195 | 326,002 | **0.60** |
| 1998 | 0 | 17 | 83 | 47 | 10 | 4 | 0 | 0 | 0 | 0 | 0 | 0 | 0 | 0 | 161 | 271,450 | **0.59** |
| 1999 | 0 | 0 | 22 | 76 | 59 | 10 | 8 | 4 | 0 | 0 | 0 | 0 | 0 | 0 | 179 | 283,661 | **0.63** |
| 2000 | 0 | 0 | 0 | 22 | 97 | 67 | 17 | 3 | 3 | 0 | 0 | 0 | 0 | 0 | 209 | 305,312 | **0.68** |
| 2001 | 0 | 0 | 0 | 0 | 29 | 92 | 44 | 17 | 2 | 1 | 0 | 0 | 0 | 0 | 185 | 260,354 | **0.71** |
| 2002 | 0 | 0 | 0 | 0 | 0 | 19 | 73 | 60 | 13 | 8 | 3 | 0 | 0 | 0 | 176 | 247,530 | **0.71** |
| 2003 | 0 | 0 | 0 | 0 | 0 | 0 | 20 | 64 | 39 | 5 | 2 | 2 | 0 | 0 | 132 | 227,070 | **0.58** |
| 2004 | 0 | 0 | 0 | 0 | 0 | 0 | 0 | 24 | 56 | 44 | 2 | 4 | 2 | 0 | 132 | 216,419 | **0.61** |
| 2005 | 0 | 0 | 0 | 0 | 0 | 0 | 0 | 0 | 18 | 35 | 40 | 4 | 1 | 2 | 100 | 205,854 | **0.49** |
| Total | 21 | 102 | 169 | 162 | 202 | 193 | 162 | 172 | 131 | 93 | 47 | 10 | 3 | 2 | 1469 |  |  |

**Table 1-1、Case number of first surgery by year of birth and year of operation (Girl)**

| Birth year | Operation year | | | | | | | | | | | |  |  | Total | Live births | incidence rate (per 1,000 live births) |
| --- | --- | --- | --- | --- | --- | --- | --- | --- | --- | --- | --- | --- | --- | --- | --- | --- | --- |
|  | 1997 | 1998 | 1999 | 2000 | 2001 | 2002 | 2003 | 2004 | 2005 | 2006 | 2007 | 2008 | 2009 | 2010 |  |  |  |
| 1997 | 19 | 76 | 62 | 17 | 6 | 1 | 0 | 0 | 0 | 0 | 0 | 0 | 0 | 0 | 181 | 155,955 | **1.16** |
| 1998 | 0 | 16 | 75 | 45 | 8 | 4 | 0 | 0 | 0 | 0 | 0 | 0 | 0 | 0 | 148 | 129,988 | **1.14** |
| 1999 | 0 | 0 | 17 | 67 | 52 | 7 | 7 | 2 | 0 | 0 | 0 | 0 | 0 | 0 | 152 | 135,619 | **1.12** |
| 2000 | 0 | 0 | 0 | 20 | 85 | 62 | 14 | 2 | 3 | 0 | 0 | 0 | 0 | 0 | 186 | 145,586 | **1.28** |
| 2001 | 0 | 0 | 0 | 0 | 25 | 84 | 39 | 15 | 2 | 1 | 0 | 0 | 0 | 0 | 166 | 124,758 | **1.33** |
| 2002 | 0 | 0 | 0 | 0 | 0 | 14 | 70 | 50 | 12 | 6 | 3 | 0 | 0 | 0 | 155 | 117,993 | **1.31** |
| 2003 | 0 | 0 | 0 | 0 | 0 | 0 | 16 | 49 | 36 | 4 | 1 | 2 | 0 | 0 | 108 | 108,086 | **1.00** |
| 2004 | 0 | 0 | 0 | 0 | 0 | 0 | 0 | 22 | 52 | 38 | 2 | 3 | 2 | 0 | 119 | 102,780 | **1.16** |
| 2005 | 0 | 0 | 0 | 0 | 0 | 0 | 0 | 0 | 15 | 32 | 34 | 2 | 1 | 1 | 85 | 98,476 | **0.86** |
| Total | 19 | 92 | 154 | 149 | 176 | 172 | 146 | 140 | 120 | 81 | 40 | 7 | 3 | 1 | 1300 |  |  |

**Table 1-2、Case number of first surgery by year of birth and year of operation (boy**)

| Birth year | Operation year | | | | | | | | | | | |  |  | Total | Live births | incidence rate (per 1,000 live births) |
| --- | --- | --- | --- | --- | --- | --- | --- | --- | --- | --- | --- | --- | --- | --- | --- | --- | --- |
|  | 1997 | 1998 | 1999 | 2000 | 2001 | 2002 | 2003 | 2004 | 2005 | 2006 | 2007 | 2008 | 2009 | 2010 |  |  |  |
| 1997 | 2 | 9 | 2 | 0 | 1 | 0 | 0 | 0 | 0 | 0 | 0 | 0 | 0 | 0 | 14 | 170,047 | **0.08** |
| 1998 | 0 | 1 | 8 | 2 | 2 | 0 | 0 | 0 | 0 | 0 | 0 | 0 | 0 | 0 | 13 | 141,462 | **0.09** |
| 1999 | 0 | 0 | 5 | 8 | 7 | 3 | 1 | 2 | 0 | 0 | 0 | 0 | 0 | 0 | 26 | 148,042 | **0.18** |
| 2000 | 0 | 0 | 0 | 2 | 12 | 5 | 3 | 1 | 0 | 0 | 0 | 0 | 0 | 0 | 23 | 159,726 | **0.14** |
| 2001 | 0 | 0 | 0 | 0 | 4 | 8 | 5 | 2 | 0 | 0 | 0 | 0 | 0 | 0 | 19 | 135,596 | **0.14** |
| 2002 | 0 | 0 | 0 | 0 | 0 | 5 | 3 | 10 | 1 | 2 | 0 | 0 | 0 | 0 | 21 | 129,537 | **0.16** |
| 2003 | 0 | 0 | 0 | 0 | 0 | 0 | 4 | 15 | 3 | 1 | 1 | 0 | 0 | 0 | 24 | 118,984 | **0.20** |
| 2004 | 0 | 0 | 0 | 0 | 0 | 0 | 0 | 2 | 4 | 6 | 0 | 1 | 0 | 0 | 13 | 113,639 | **0.11** |
| 2005 | 0 | 0 | 0 | 0 | 0 | 0 | 0 | 0 | 3 | 3 | 6 | 2 | 0 | 1 | 15 | 107,378 | **0.14** |
| Total | 2 | 10 | 15 | 12 | 26 | 21 | 16 | 32 | 11 | 12 | 7 | 3 | 0 | 1 | 168 |  |  |


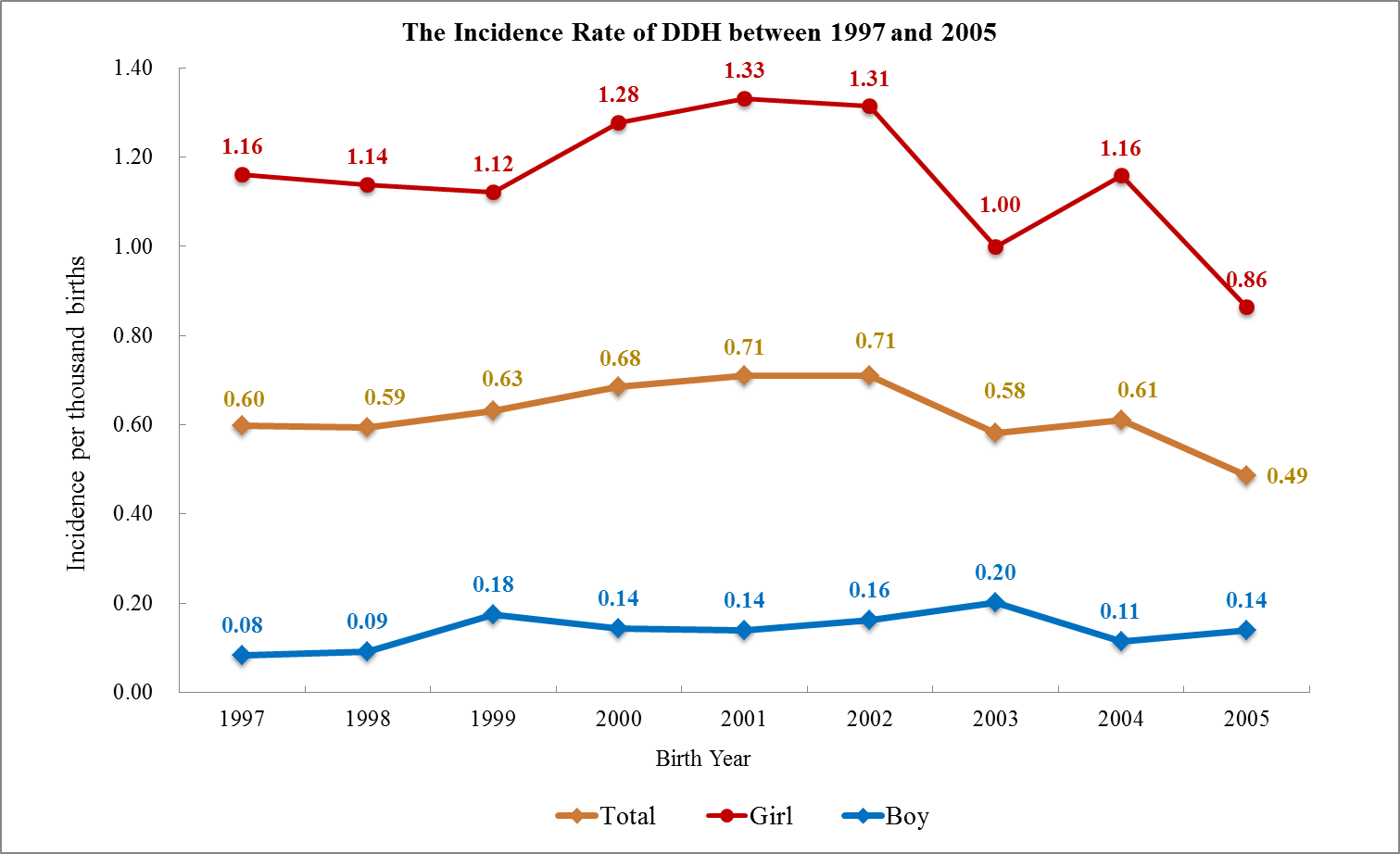


Table 2-1、Case number of first surgery by birth year and age of operation (Total)

| Birth year | Operation age | | | | | | | | Total |
| --- | --- | --- | --- | --- | --- | --- | --- | --- | --- |
|  | ≦3 months | 4~6months | 7~9 months | 10~12months | 1~2 years | 2~3 years | 3~4 years | 4~5 years |  |
| 1997 | 7 | 10 | 13 | 9 | 116 | 23 | 15 | 2 | 195 |
| 1998 | 12 | 6 | 15 | 4 | 100 | 15 | 6 | 3 | 161 |
| 1999 | 8 | 27 | 10 | 7 | 96 | 15 | 7 | 9 | 179 |
| 2000 | 7 | 21 | 23 | 10 | 110 | 25 | 8 | 5 | 209 |
| 2001 | 10 | 14 | 24 | 7 | 98 | 26 | 4 | 2 | 185 |
| 2002 | 8 | 14 | 11 | 11 | 97 | 20 | 9 | 6 | 176 |
| 2003 | 1 | 17 | 12 | 9 | 73 | 15 | 1 | 4 | 132 |
| 2004 | 8 | 14 | 14 | 5 | 72 | 12 | 4 | 3 | 132 |
| 2005 | 7 | 6 | 8 | 6 | 53 | 15 | 2 | 3 | 100 |
| Total | 68 | 129 | 130 | 68 | 815 | 166 | 56 | 37 | 1469 |

Table 2-2、Incidence rate of operation age group by birth year (Total)

| Birth year | Operation age | | | | | | | |
| --- | --- | --- | --- | --- | --- | --- | --- | --- |
|  | ≦3 months | 4~6months | 7~9 months | 10~12months | 1~2 years | 2~3 years | 3~4 years | 4~5 years |
| 1997 | 0.021 | 0.031 | 0.040 | 0.028 | 0.356 | 0.071 | 0.046 | 0.006 |
| 1998 | 0.044 | 0.022 | 0.055 | 0.015 | 0.368 | 0.055 | 0.022 | 0.011 |
| 1999 | 0.028 | 0.095 | 0.035 | 0.025 | 0.338 | 0.053 | 0.025 | 0.032 |
| 2000 | 0.023 | 0.069 | 0.075 | 0.033 | 0.360 | 0.082 | 0.026 | 0.016 |
| 2001 | 0.038 | 0.054 | 0.092 | 0.027 | 0.376 | 0.100 | 0.015 | 0.008 |
| 2002 | 0.032 | 0.057 | 0.044 | 0.044 | 0.392 | 0.081 | 0.036 | 0.024 |
| 2003 | 0.004 | 0.075 | 0.053 | 0.040 | 0.321 | 0.066 | 0.004 | 0.018 |
| 2004 | 0.037 | 0.065 | 0.065 | 0.023 | 0.333 | 0.055 | 0.018 | 0.014 |
| 2005 | 0.034 | 0.029 | 0.039 | 0.029 | 0.257 | 0.073 | 0.010 | 0.015 |


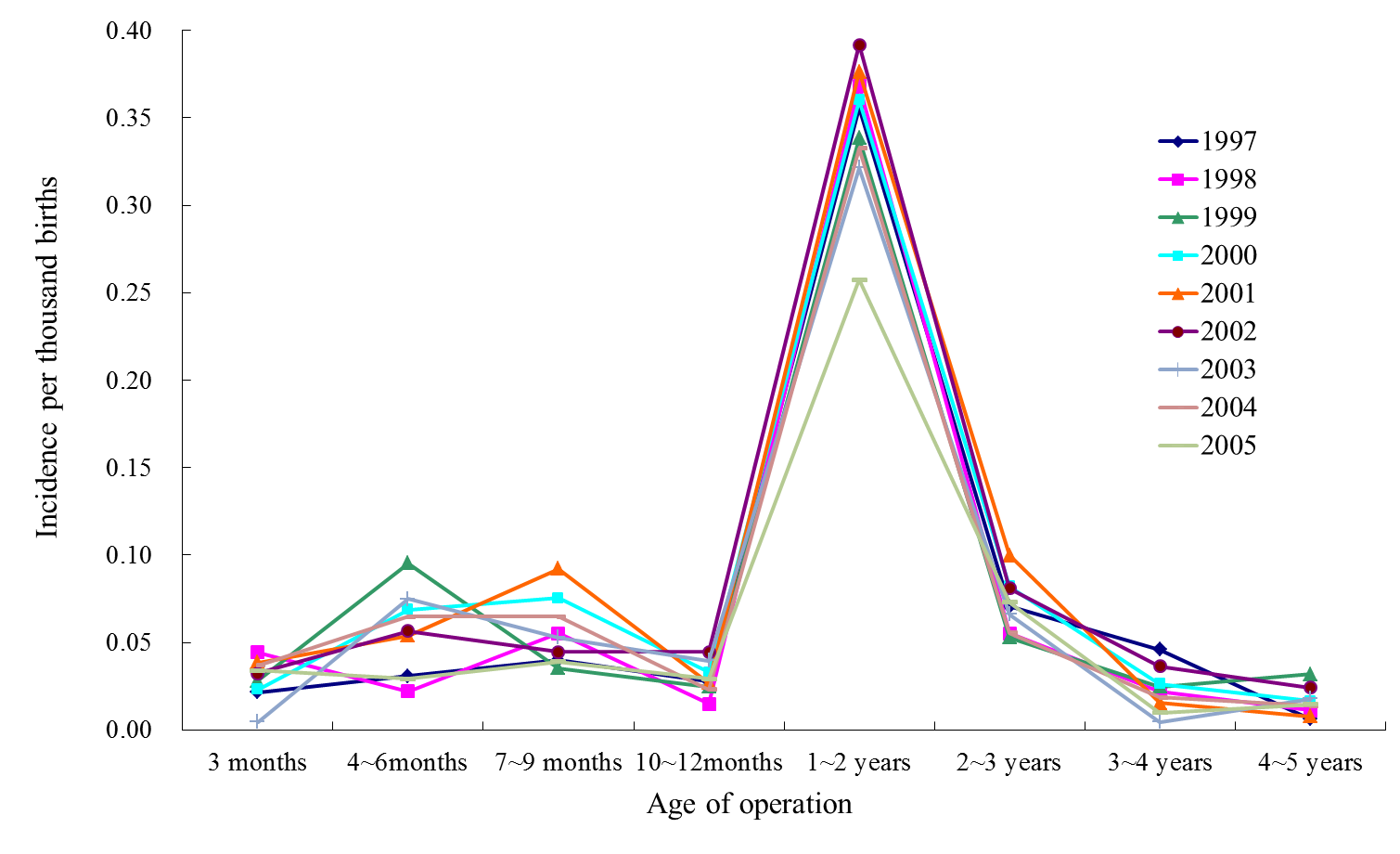


Table 2-1-1、Case number of first surgery by birth year and age of operation (Girl)

| Birth year | Operation age | | | | | | | | Total |
| --- | --- | --- | --- | --- | --- | --- | --- | --- | --- |
|  | ≦3 months | 4~6months | 7~9 months | 10~12months | 1~2 years | 2~3 years | 3~4 years | 4~5 years |  |
| 1997 | 7 | 9 | 10 | 9 | 107 | 23 | 14 | 2 | 181 |
| 1998 | 11 | 6 | 15 | 4 | 90 | 14 | 5 | 3 | 148 |
| 1999 | 5 | 25 | 10 | 6 | 82 | 12 | 5 | 7 | 152 |
| 2000 | 7 | 20 | 21 | 7 | 100 | 19 | 7 | 5 | 186 |
| 2001 | 7 | 12 | 23 | 6 | 90 | 22 | 4 | 2 | 166 |
| 2002 | 5 | 13 | 9 | 11 | 85 | 19 | 7 | 6 | 155 |
| 2003 | 0 | 16 | 8 | 6 | 61 | 13 | 1 | 3 | 108 |
| 2004 | 7 | 13 | 12 | 5 | 64 | 12 | 3 | 3 | 119 |
| 2005 | 7 | 4 | 6 | 6 | 49 | 10 | 1 | 2 | 85 |
| Total | 56 | 118 | 114 | 60 | 728 | 144 | 47 | 33 | 1300 |

Table 2-2-1、Incidence rate of operation age group by birth year (Girl)

| Birth year |  |  | | Operation age | | | | | | |
| --- | --- | --- | --- | --- | --- | --- | --- | --- | --- | --- |
|  | ≦3 months | | 4~6months | | 7~9 months | 10~12months | 1~2 years | 2~3 years | 3~4 years | 4~5 years |
| 1997 | 0.045 | | 0.058 | | 0.064 | 0.058 | 0.686 | 0.147 | 0.090 | 0.013 |
| 1998 | 0.085 | | 0.046 | | 0.115 | 0.031 | 0.692 | 0.108 | 0.038 | 0.023 |
| 1999 | 0.037 | | 0.184 | | 0.074 | 0.044 | 0.605 | 0.088 | 0.037 | 0.052 |
| 2000 | 0.048 | | 0.137 | | 0.144 | 0.048 | 0.687 | 0.131 | 0.048 | 0.034 |
| 2001 | 0.056 | | 0.096 | | 0.184 | 0.048 | 0.721 | 0.176 | 0.032 | 0.016 |
| 2002 | 0.042 | | 0.110 | | 0.076 | 0.093 | 0.720 | 0.161 | 0.059 | 0.051 |
| 2003 | 0.000 | | 0.148 | | 0.074 | 0.056 | 0.564 | 0.120 | 0.009 | 0.028 |
| 2004 | 0.068 | | 0.126 | | 0.117 | 0.049 | 0.623 | 0.117 | 0.029 | 0.029 |
| 2005 | 0.071 | | 0.041 | | 0.061 | 0.061 | 0.498 | 0.102 | 0.010 | 0.020 |


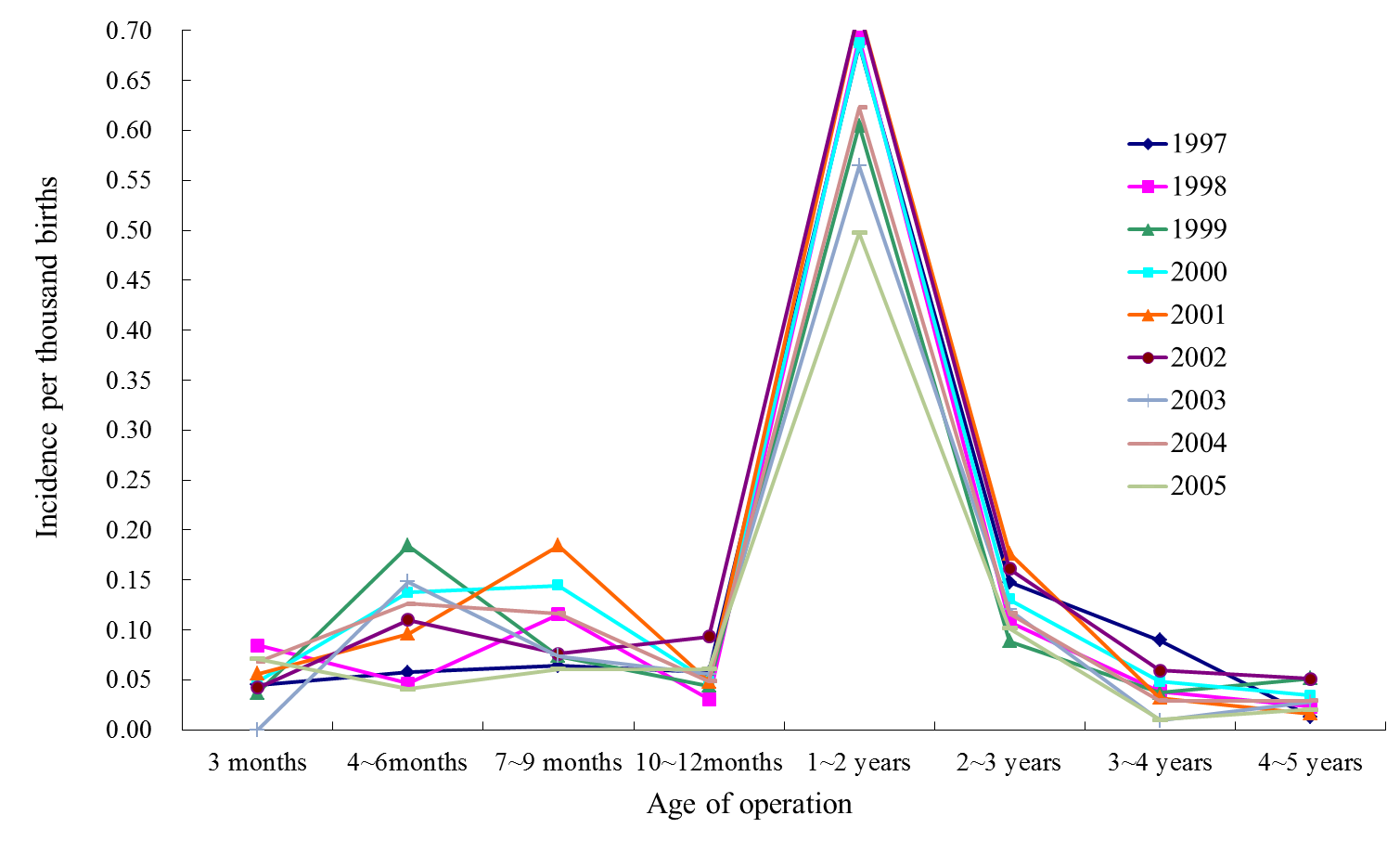


Table 2-1-2、Case number of first surgery by year of birth and age of operation (Boy)

| Birth year | Operation age | | | | | | | | Total |
| --- | --- | --- | --- | --- | --- | --- | --- | --- | --- |
|  | ≦3 months | 4~6months | 7~9 months | 10~12months | 1~2 years | 2~3 years | 3~4 years | 4~5 years |  |
| 1997 | 0 | 1 | 3 | 0 | 9 | 0 | 1 | 0 | 14 |
| 1998 | 1 | 0 | 0 | 0 | 10 | 1 | 1 | 0 | 13 |
| 1999 | 3 | 2 | 0 | 1 | 13 | 3 | 2 | 2 | 26 |
| 2000 | 0 | 1 | 2 | 3 | 10 | 6 | 1 | 0 | 23 |
| 2001 | 3 | 2 | 1 | 1 | 8 | 4 | 0 | 0 | 19 |
| 2002 | 3 | 1 | 2 | 0 | 12 | 1 | 2 | 0 | 21 |
| 2003 | 1 | 1 | 4 | 3 | 12 | 2 | 0 | 1 | 24 |
| 2004 | 1 | 1 | 2 | 0 | 8 | 0 | 1 | 0 | 13 |
| 2005 | 0 | 2 | 2 | 0 | 4 | 5 | 1 | 1 | 15 |
| Total | 12 | 11 | 16 | 8 | 86 | 22 | 9 | 4 | 168 |

Table 2-2-2、Incidence rate of operation age group by birth year (Boy)

| Birth year | Operation age | | | | | | | | Total |
| --- | --- | --- | --- | --- | --- | --- | --- | --- | --- |
|  | ≦3 months | 4~6months | 7~9 months | 10~12months | 1~2 years | 2~3 years | 3~4 years | 4~5 years |  |
| 1997 | 0.000 | 0.006 | 0.018 | 0.000 | 0.053 | 0.000 | 0.006 | 0.000 | 0.000 |
| 1998 | 0.007 | 0.000 | 0.000 | 0.000 | 0.071 | 0.007 | 0.007 | 0.000 | 0.007 |
| 1999 | 0.020 | 0.014 | 0.000 | 0.007 | 0.088 | 0.020 | 0.014 | 0.014 | 0.020 |
| 2000 | 0.000 | 0.006 | 0.013 | 0.019 | 0.063 | 0.038 | 0.006 | 0.000 | 0.000 |
| 2001 | 0.022 | 0.015 | 0.007 | 0.007 | 0.059 | 0.029 | 0.000 | 0.000 | 0.022 |
| 2002 | 0.023 | 0.008 | 0.015 | 0.000 | 0.093 | 0.008 | 0.015 | 0.000 | 0.023 |
| 2003 | 0.008 | 0.008 | 0.034 | 0.025 | 0.101 | 0.017 | 0.000 | 0.008 | 0.008 |
| 2004 | 0.009 | 0.009 | 0.018 | 0.000 | 0.070 | 0.000 | 0.009 | 0.000 | 0.009 |
| 2005 | 0.000 | 0.019 | 0.019 | 0.000 | 0.037 | 0.047 | 0.009 | 0.009 | 0.000 |
| Total | 0.000 | 0.006 | 0.018 | 0.000 | 0.053 | 0.000 | 0.006 | 0.000 | 0.000 |


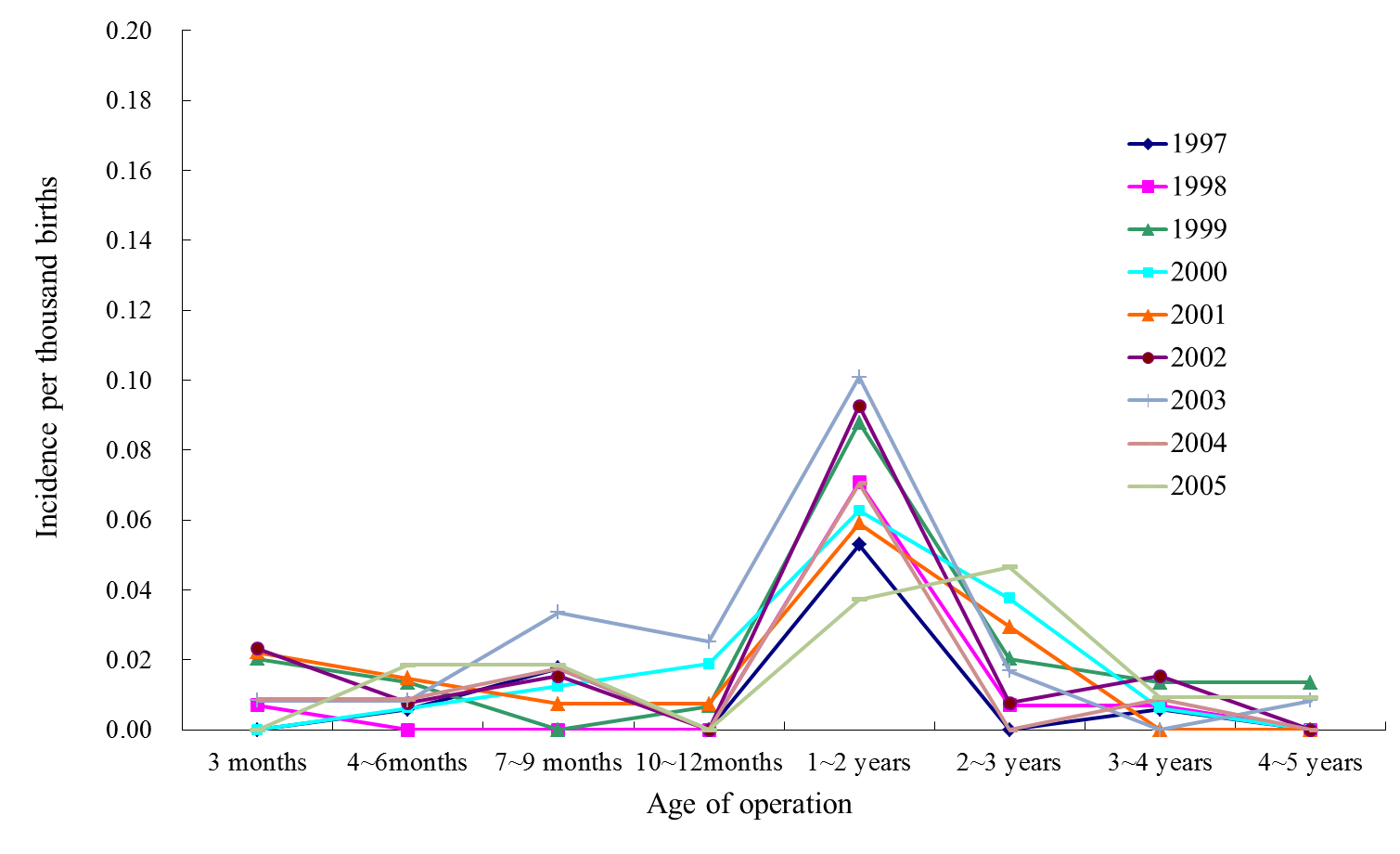


Table 3-1、Case number of first surgery by year of birth and type of operation (Total)

| Birth year | Operation type | | Total |
| --- | --- | --- | --- |
|  | major | minor |  |
| 1997 | 138 | 57 | 195 |
| 1998 | 100 | 61 | 161 |
| 1999 | 114 | 65 | 179 |
| 2000 | 125 | 84 | 209 |
| 2001 | 116 | 69 | 185 |
| 2002 | 116 | 60 | 176 |
| 2003 | 85 | 47 | 132 |
| 2004 | 78 | 54 | 132 |
| 2005 | 67 | 33 | 100 |
| Total | 939 | 530 | 1469 |

Table 3-2 Incidence rate of operation type group by birth year (Total)

| Birth year | Operation type | |
| --- | --- | --- |
|  | major | minor |
| 1997 | 0.42 | 0.17 |
| 1998 | 0.37 | 0.22 |
| 1999 | 0.40 | 0.23 |
| 2000 | 0.41 | 0.28 |
| 2001 | 0.45 | 0.27 |
| 2002 | 0.47 | 0.24 |
| 2003 | 0.37 | 0.21 |
| 2004 | 0.36 | 0.25 |
| 2005 | 0.33 | 0.16 |


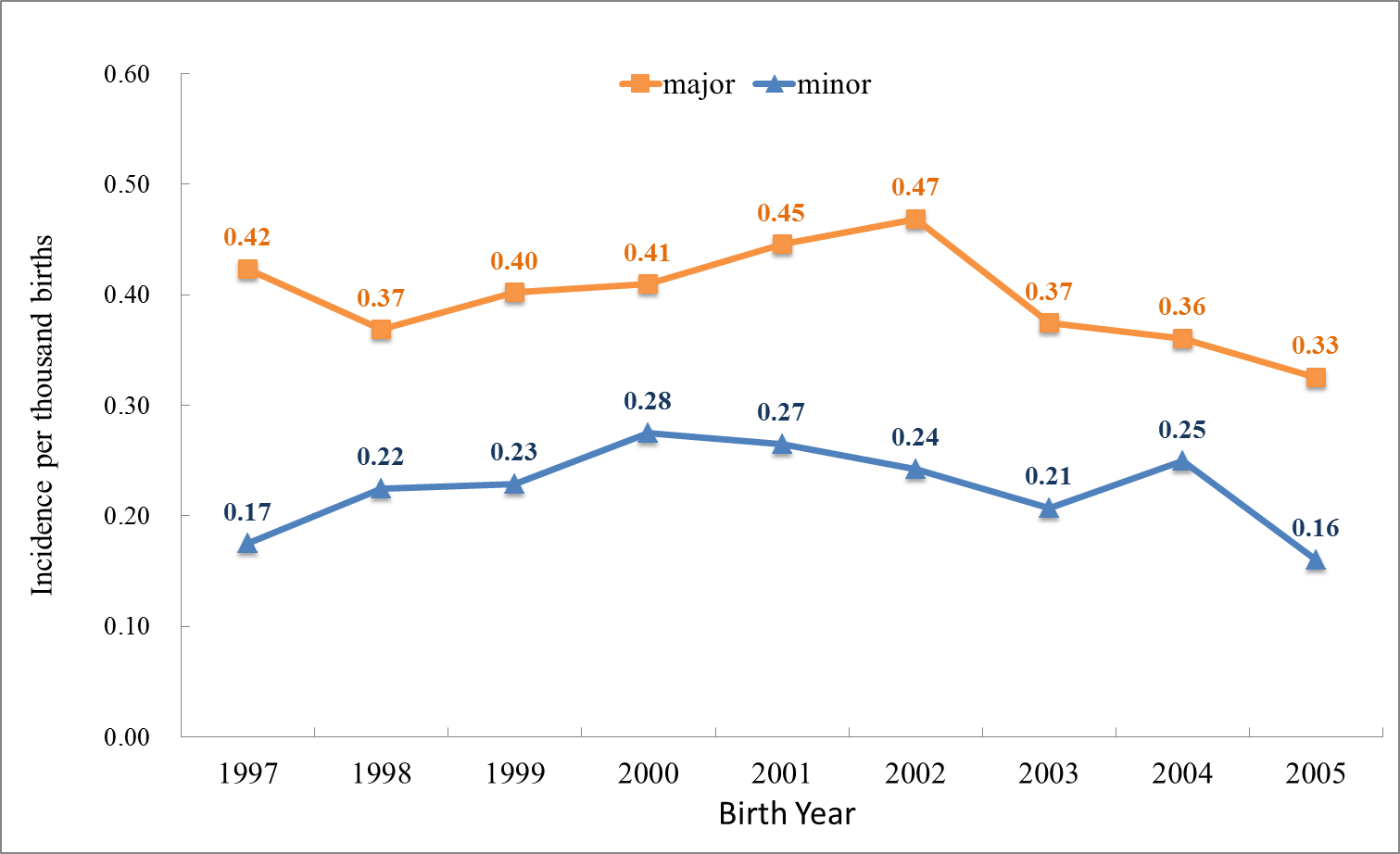


Table 3-1-1 Case number of first surgery by year of birth and type of operation (Girl)

| Birth year | Operation type | | Total |
| --- | --- | --- | --- |
|  | major | minor |  |
| 1997 | 131 | 50 | 181 |
| 1998 | 90 | 58 | 148 |
| 1999 | 96 | 56 | 152 |
| 2000 | 109 | 77 | 186 |
| 2001 | 104 | 62 | 166 |
| 2002 | 104 | 51 | 155 |
| 2003 | 69 | 39 | 108 |
| 2004 | 71 | 48 | 119 |
| 2005 | 57 | 28 | 85 |
| Total | 831 | 469 | 1300 |

Table 3-2-1 Incidence rate of operation type group by birth year (Girl)

| Birth Year | Operation type | |
| --- | --- | --- |
|  | major | minor |
| 1997 | 0.84 | 0.32 |
| 1998 | 0.69 | 0.45 |
| 1999 | 0.71 | 0.41 |
| 2000 | 0.75 | 0.53 |
| 2001 | 0.83 | 0.50 |
| 2002 | 0.88 | 0.43 |
| 2003 | 0.64 | 0.36 |
| 2004 | 0.69 | 0.47 |
| 2005 | 0.58 | 0.28 |


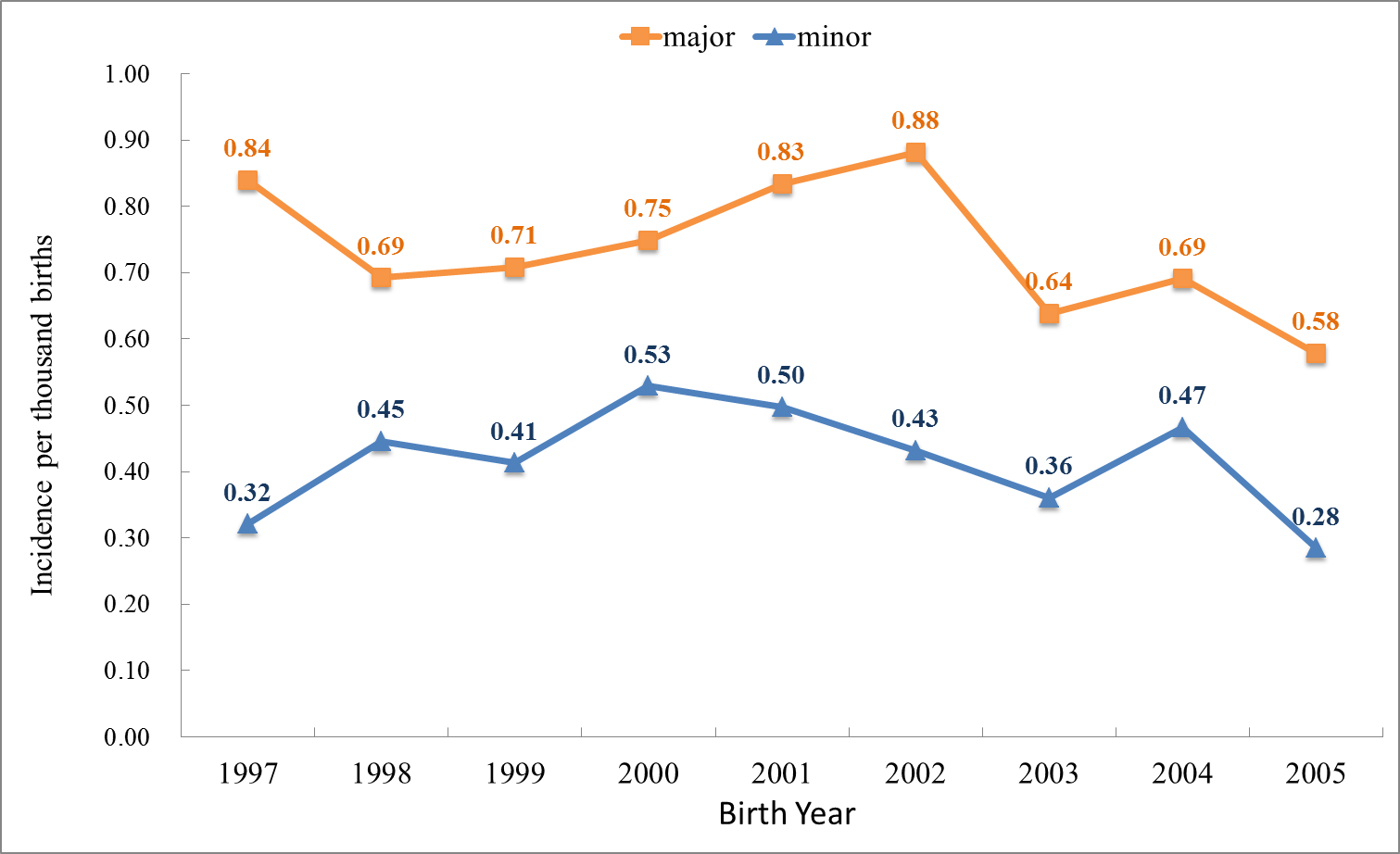


Table 3-1-2 Case number of first surgery by year of birth and type of operation (Boy)

| Birth year | Operation type | | Total |
| --- | --- | --- | --- |
|  | major | minor |  |
| 1997 | 7 | 7 | 14 |
| 1998 | 10 | 3 | 13 |
| 1999 | 18 | 8 | 26 |
| 2000 | 16 | 7 | 23 |
| 2001 | 12 | 7 | 19 |
| 2002 | 12 | 9 | 21 |
| 2003 | 16 | 8 | 24 |
| 2004 | 7 | 6 | 13 |
| 2005 | 10 | 5 | 15 |
| Total | 108 | 60 | 168 |

Table 3-2-2 Incidence rate of operation type group by birth year (Boy)

| Birth year | Operation type | |
| --- | --- | --- |
|  | major | minor |
| 1997 | 0.04 | 0.04 |
| 1998 | 0.07 | 0.02 |
| 1999 | 0.12 | 0.05 |
| 2000 | 0.10 | 0.04 |
| 2001 | 0.09 | 0.05 |
| 2002 | 0.09 | 0.07 |
| 2003 | 0.13 | 0.07 |
| 2004 | 0.06 | 0.05 |
| 2005 | 0.09 | 0.05 |


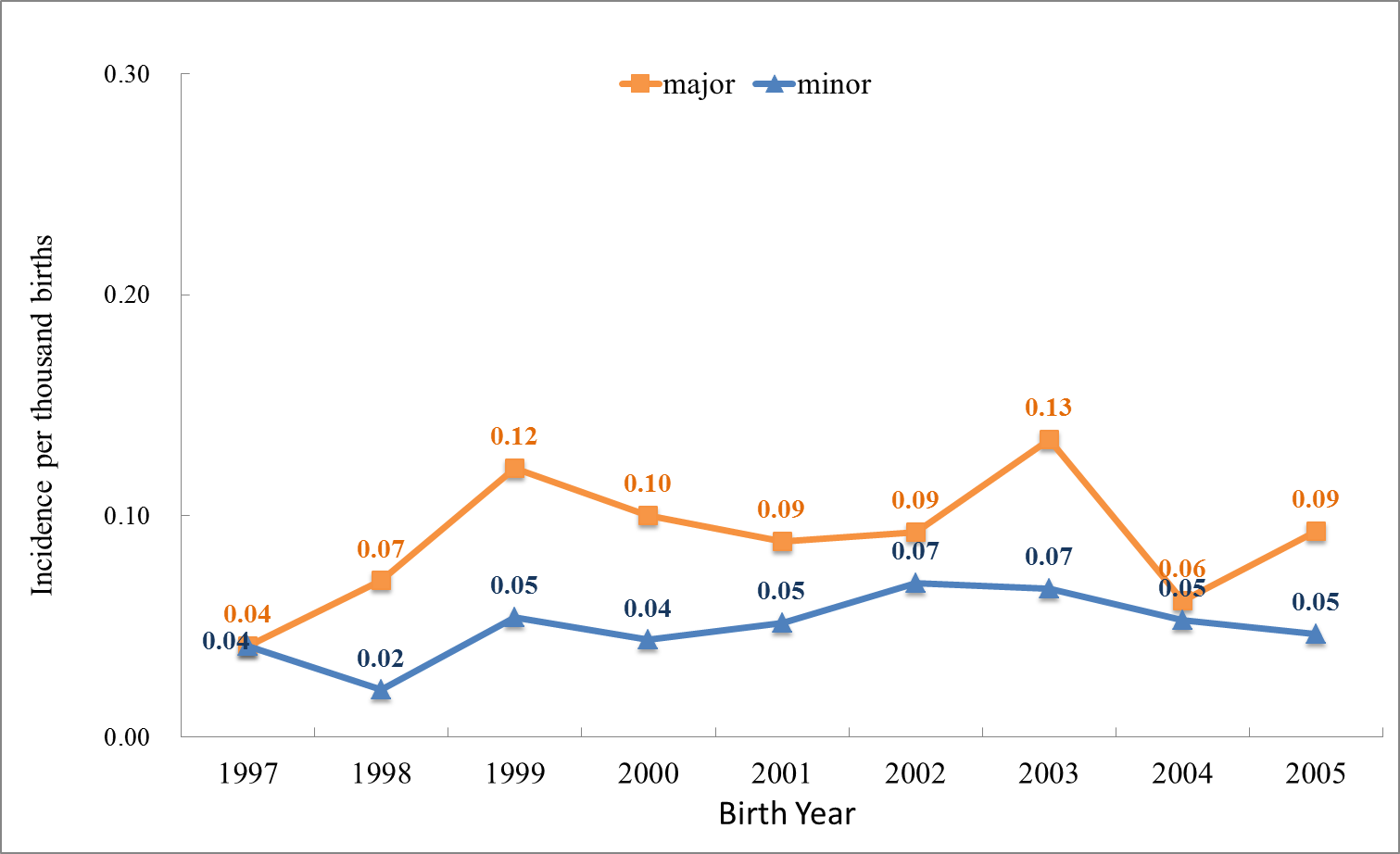


Table 4. Case number of first surgery by year of birth and geographic area (Total)

|  | Northern | Central | Southern | Others | Total |
| --- | --- | --- | --- | --- | --- |
| 1997 | 106 | 41 | 47 | 1 | 195 |
| 1998 | 101 | 27 | 33 | 0 | 161 |
| 1999 | 85 | 47 | 46 | 1 | 179 |
| 2000 | 105 | 51 | 52 | 1 | 209 |
| 2001 | 95 | 45 | 42 | 3 | 185 |
| 2002 | 95 | 34 | 46 | 1 | 176 |
| 2003 | 62 | 28 | 41 | 1 | 132 |
| 2004 | 53 | 48 | 31 | 0 | 132 |
| 2005 | 43 | 31 | 26 | 0 | 100 |
| Total | 745 | 352 | 364 | 8 | 1469 |

Table 5. Case number of DDH patients by year of birth and medical setting (Total)

|  | Medical center | Regional hospital | District hospital | Clinics and others | Total |
| --- | --- | --- | --- | --- | --- |
| 1997 | 116 | 69 | 10 | 0 | 195 |
| 1998 | 89 | 67 | 5 | 0 | 161 |
| 1999 | 111 | 66 | 2 | 0 | 179 |
| 2000 | 151 | 51 | 7 | 0 | 209 |
| 2001 | 138 | 39 | 8 | 0 | 185 |
| 2002 | 143 | 32 | 1 | 0 | 176 |
| 2003 | 105 | 26 | 1 | 0 | 132 |
| 2004 | 98 | 30 | 4 | 0 | 132 |
| 2005 | 75 | 22 | 2 | 1 | 100 |
| Total | 1026 | 402 | 40 | 1 | 1469 |
